# Yes/No Quantitative Analysis of Single-Stranded Oligonucleotides with Lateral Flow Assays

**DOI:** 10.3390/bios16090465

**Published:** 2026-08-26

**Authors:** Niusha Hassandoost, Leslie Munoz, Kerrigan Kotecki, Irina V. Nesterova

**Affiliations:** Department of Chemistry and Biochemistry, Northern Illinois University, DeKalb, IL 60115, USA

**Keywords:** quantitative analysis, molecular diagnostics, lateral flow assay

## Abstract

Accessible molecular diagnostics is fundamental to effective healthcare. While most current point-of-care devices detect only the presence of a molecular biomarker(s), biomarker quantification can be equally important for decision-making on disease treatment and containment. Here, we present a diagnostic platform that enables the equipment-free quantification of molecular biomarkers with the simplicity of a binary (yes/no) readout. This capability is achieved by integrating a stoichiometric quantitative approach with widely available and easy-to-use lateral flow dipsticks. To implement the approach, we engineer negative cooperativity into target–probe binding interactions for oligonucleotide targets as a model system. The resulting threshold-based semi-quantitative assay with lateral flow dipsticks quantifies targets in the low-nanomolar range and operates reliably in complex biological backgrounds. A key advantage of this platform is its potential adaptability to new and emerging targets: repurposing will require only reagent redesign, without the need for additional fabrication.

## 1. Introduction

Reliable, accessible molecular diagnostics is essential for both public and individual health. Communities and individuals, ranging from affluent urban centers to under-resourced rural regions, depend on these tools to control and contain epidemics, diagnose time-sensitive conditions, and supplement or even replace centralized clinical laboratories. Recent epidemics and pandemics have accelerated the development of lateral flow assays (LFAs), underscoring the practical importance and life-saving impact of rapid and accurate diagnostics delivered where it is needed: at or near the patient [1,2].

Two kinds of information about a biomarker can carry diagnostic significance: qualitative and quantitative. While qualitative information (whether a biomarker is present) is often sufficient to assess a condition, quantitative assessment (how much of the biomarker is present) can be a decisive factor in many circumstances related to personal and public health [3]. Therefore, the accessibility of diagnostic devices that provide fast and reliable quantitative results is vital in situations ranging from epidemic breakouts to routine healthcare needs [4,5,6,7,8,9].

Currently, quantitative molecular diagnostics relies on several well-established platforms. The “workhorses”, qPCR and ELISA, provide reliable results for nucleic acids [10,11] and protein targets [12,13,14], respectively, with qPCRs being theoretically able to detect a single copy of a target. However, both technologies require equipped facilities and trained personnel, which limits their reach in settings with direct access to appropriate clinical labs. Accessibility of quantitative molecular diagnostics in true point-of-care (POC), do-it-yourself (DIY), and limited-resource (LRS) settings remainslimited [15].

The World Health Organization defines the key characteristics of a practically meaningful POC/DIY/LRS-accessible diagnostic platform using the REASSURED criteria (i.e., real-time, ease of specimen collection, affordable, sensitive, specific, user-friendly, robust and rapid, equipment-free, deliverable) [16,17]. Therefore, all steps involved in the analysis workflow (e.g., biomarker isolation, separation, amplification, signal transduction) must comply with the REASSURED requirements.

Among existing technologies, lateral flow assays (LFAs) come closest to complying with the REASSURED criteria [18]. Two attributes are essential to their POC/DIY/LRS success: (i) an equipment-free, easy-to-perform format and (ii) a clear, binary yes/no (Y/N) readout that is simple to interpret. Together, these two features empower molecular diagnostics’ proliferation beyond conventional laboratory environments.

Over the last decade, LFAs have extended into quantitative analysis [18,19], with three major working principles: (i) precise control of recognition element quantities deposited at the strip fabrication stage [20,21,22,23,24] and/or (ii) introducing dedicated signal reader hardware or software [19,21,24,25,26,27,28,29,30,31] or (iii) the naked-eye interpretation of color intensity or hue [32]. The first two approaches add costs, jeopardizing the “affordable” and “deliverable” elements of the REASSURED criteria. In addition, prefabricated devices are difficult to adapt rapidly to emerging pathogens. While being equipment-free, color/hue observation is very subjective. Collectively, the manufacturing stringency, interpretation uncertainty, and dependence on auxiliary instrumentation motivate the search for more accessible quantitative LFA strategies [19,33].

We hypothesize that the reliance on calibration-based quantitation complicates LFAs’ accessibility. Another quantitative analysis approach, stoichiometry, on the other hand, can yield a quantitative result (i.e., a numerical value) in a binary Y/N format. However, stoichiometry’s reach in biomolecular analysis is very limited [34] owing to the lack of general methodologies for transducing the equivalence point while maintaining the required specificity.

**Figure 1:**
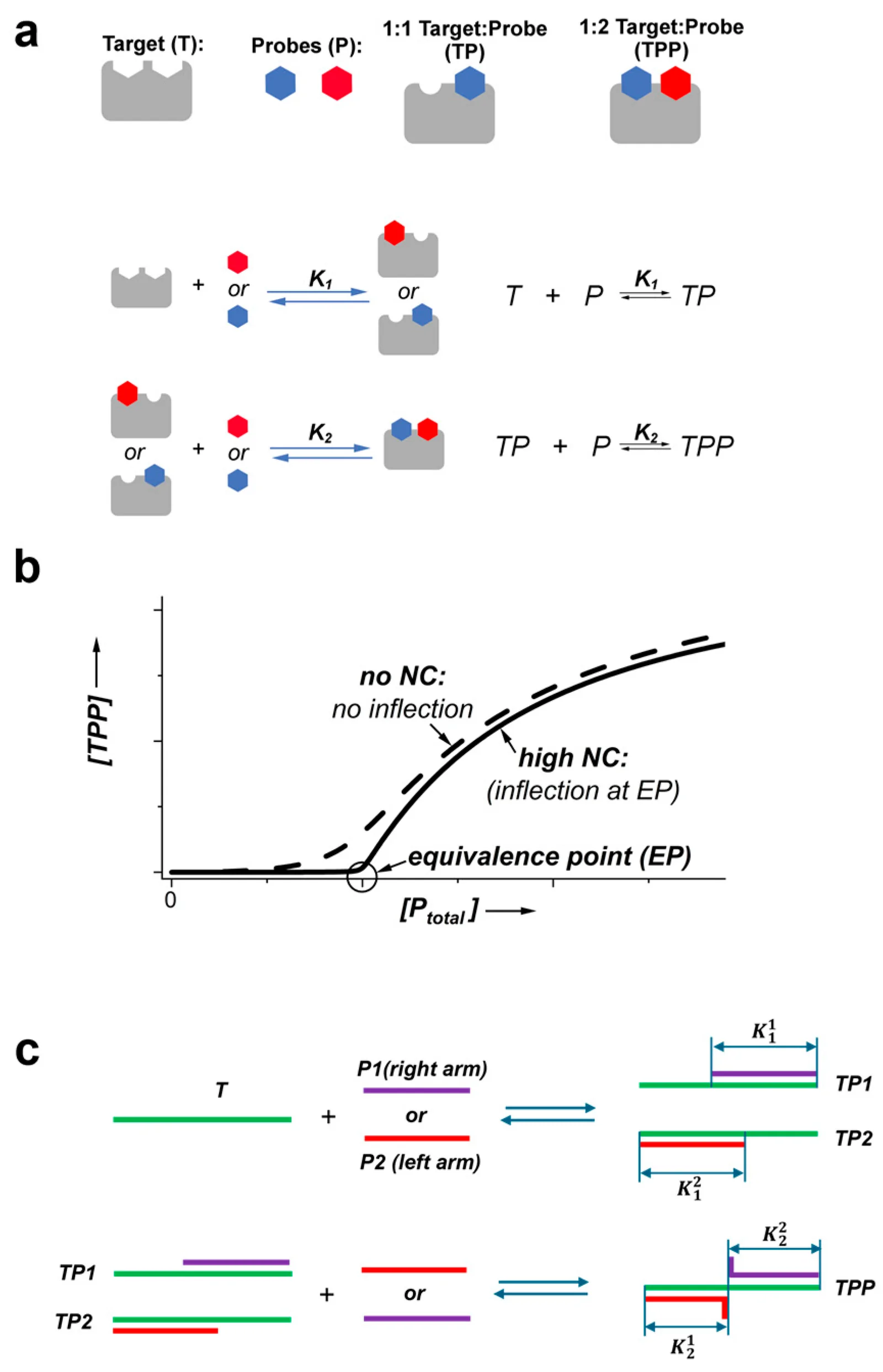
When negative cooperativity is engineered into target–probe interactions, the first binding event (formation of TP) weakens the second binding event (formation of TPP) (**a**). If the concentration of TPP as a function of the total probe concentration (with equimolar amounts in the case of different probes) is monitored, the curve will have an inflection point at the stoichiometric equivalence point in the case of negative cooperativity (solid) vs. binding conditions without NC (dashed) (**b**). Equations used to plot the curves are derived in ref. [41]. (**c**) Negative cooperativity can be engineered into oligonucleotide target–probe interactions through the incorporation of binding overlaps.

To enable the stoichiometric quantitative analysis of biomolecules, we have recently proposed to identify the equivalence point by engineering negative cooperativity into target–probe binding. In molecular biology, negative cooperativity (NC) describes the phenomenon in which the binding of one ligand to its receptor reduces the affinity for subsequent ligand–receptor interactions [35,36,37,38,39] (Figure 1a). Furthermore, when ligands bind a multimeric receptor with reasonably high affinity—and, therefore, can be appreciably depleted by the binding—NC produces a response with a clearly identifiable inflection at the stoichiometric equivalence point (Figure 1b) [38].

Here, we transform the negative cooperativity-driven stoichiometric platform into a binary Y/N threshold-based semi-quantitative analysis with simple, generic, commercially available lateral flow strips. The system yields a signal only at or above the stoichiometric equivalence point, yielding an easy-to-interpret Y/N readout. In the future, the platform will be tunable towards evolving and emerging pathogens through reagent modifications, without the need for hardware changes or refabrication. As a proof-of-concept demonstration, we developed and characterized an LFA-based platform for the quantitative analysis of nucleic acid targets and demonstrated its suitability for low-nanomolar oligonucleotide quantities in the presence of biological environments. In the reported work, we specifically focus on ssDNA as a proof-of-concept model for diagnostically relevant viral and bacterial DNA and RNAs [40].

## 2. Materials and Methods

### 2.1. Reagents and Materials

All reagents (“Bioreagent” grade or above) were sourced from established commercial sources (e.g., Thermo Fisher Scientific (Waltham, MA, USA), Sigma Aldrich (St. Louis, MO, USA)). Lateral flow assay (LFA) kits consisting of dipsticks, running buffer, and sample dilution buffer (SDB) were purchased from Cytodiagnostics, Inc. (Burlington, ON, Canada; part number LF-019-10). Gibco’s heat-inactivated fetal bovine serum and SybrGold staining dye were obtained from Thermo Fisher Scientific. A 30% acrylamide/bis solution 19:1 (5% crosslinker) used for gel preparation was obtained from Bio-Rad Laboratories (Hercules, CA, USA). The TriDye Ultra Low Range DNA Ladder was purchased from New England Biolabs (Ipswich, MA, USA).

All oligonucleotides and nuclease-free water were purchased from Integrated DNA Technologies (IDT) (Coralville, IA, USA). Oligonucleotide sequences are summarized in Table S1. Biotin- and FAM-labeled oligos were obtained as HPLC-purified; unlabeled ones were obtained as purified by desalting. All oligonucleotides were reconstituted to 100 μM stocks with nuclease-free water.

The hybridization buffer, consisting of 500 mM NaCl, 10 mM Tris-HCl, and 1 mM EDTA (pH 8.0), was prepared in-house.

### 2.2. UV–Vis Measurements

Absorbances were measured using a Cary 4000 UV–vis spectrometer (Agilent Technologies, Santa Clara, CA, USA). The actual concentration of oligonucleotide stocks was established from 260 nm absorptions using extinction coefficients provided by IDT.

### 2.3. Sample Preparation

Before hybridization, 1 µM oligonucleotide solutions prepared in hybridization buffer (500 mM NaCl, 10 mM Tris-HCl, and 1 mM EDTA (pH 8.0)) were denatured at 95 °C for 5 min, followed by cooling to room temperature overnight. Analysis samples were prepared by combining the denatured and cooled target and probe solutions at defined molar ratios in hybridization buffer. The hybridization samples were prepared and, after mixing, incubated for 30–120 min at room temperature.

### 2.4. Size Exclusion Chromatography (SEC)

**Figure 2:**
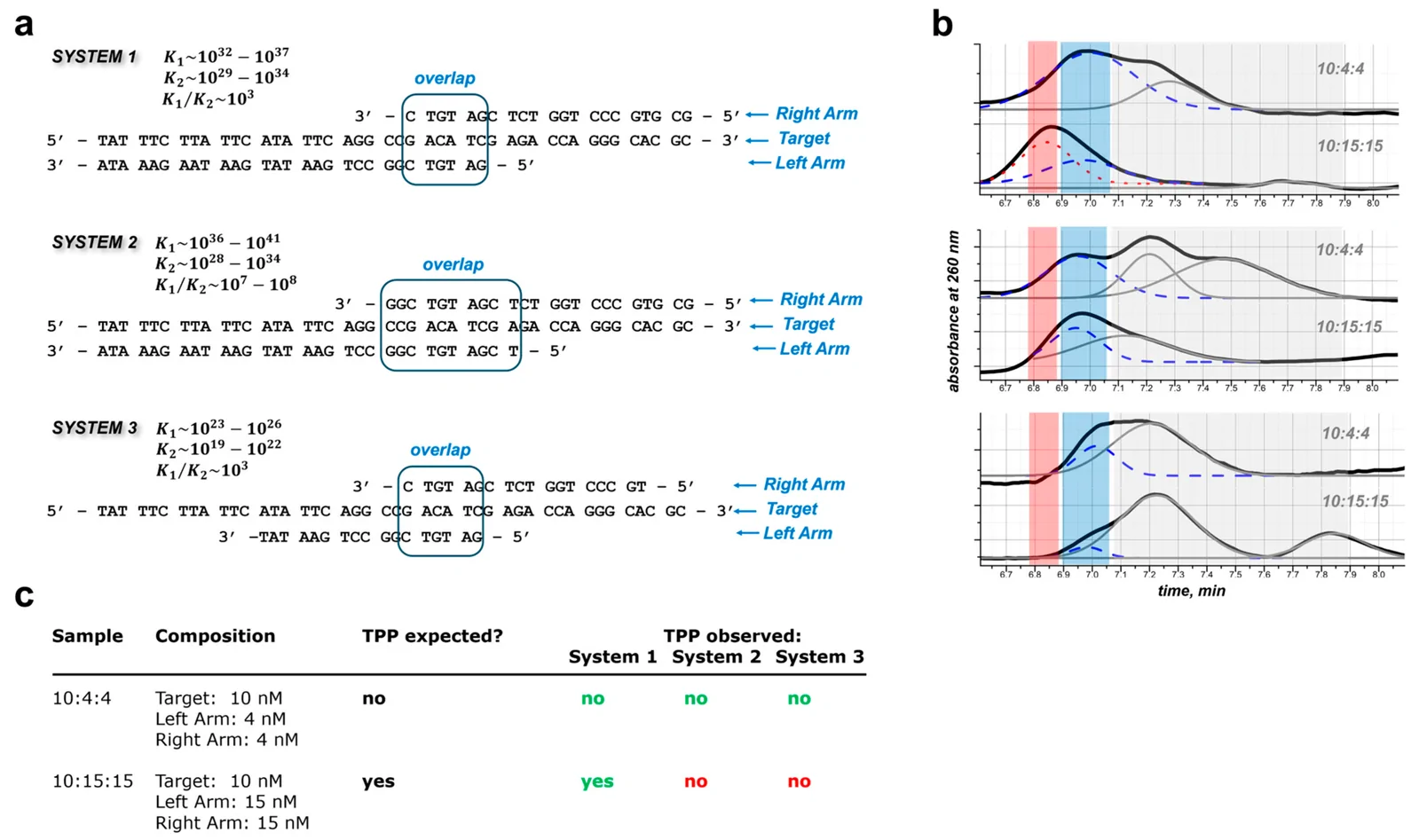
(**a**) Varying probe length and overlap extent enables the integration of negative cooperativity with a range of *K*_1_, *K*_2_, and *K*_1_/*K*_2_ into oligonucleotide target–probe systems. (**b**) SEC traces for Systems 1 to 3 (top to bottom) indicate no TPP formation below the equivalence point (samples 10:4:4). Above the equivalence point (sample 10:15:15), TPP is detected only in System 1. Pink shade (~6.78–6.88 min) indicates retention time range for expected TPP (~6.78–6.88 min); blue shade (~6.90–7.05 min) correlates with retention time established for duplexes (TPs); and grey shade (~7.08–7.89 min) is the established retention time range for single-stranded T, P1, and P2. All individual chromatograms are included in Figure S1; additional confirmation of System 1 performance via non-denaturing gel electrophoresis is included in Figure S10. Blue dotted line and grey lines are multiple-peak fits, solid black line is observed chromatogram. (**c**) Summary of SEC results indicates that System 1 best satisfies the criteria for Y/N reporting of the equivalence point.

The formation of target–probe (TP) and target–probe–probe (TPP) complexes (Figure 2 and Figure S1) was interrogated via SEC. SEC analysis was performed on an isocratic HPLC system equipped with a UV–vis detector (Shimadzu Corporation (Kyoto, Japan)). An Acclaim™ SEC-300 Å column (5 μm particle size, 4.6 × 300 mm) acquired from Thermo Fisher Scientific was used for separations. Hybridization buffer (500 mM NaCl, 10 mM Tris-HCl, and 1 mM EDTA (pH 8.0)) was used as the mobile phase. Chromatographic separation was carried out for 13 min at a flow rate of 0.35 mL/min. Elution profiles were monitored at 260 nm. Peak positions corresponding to individual components (target, probes, TP duplexes) were established from injections of the corresponding components.

### 2.5. Non-Denaturing Polyacrylamide Gel Electrophoresis

System 1’s performance was confirmed via non-denaturing polyacrylamide gel electrophoresis (Figure S10). The separation was carried out on 20% polyacrylamide in 0.5 × TBE running buffer at ~4 °C for 3 h at 150 V. Following electrophoresis, the gels were stained with SYBRGold for 20 min and visualized with a Typhoon 5 biomolecular imager (Cytiva, Marlborough, MA, USA).

### 2.6. Lateral Flow Assay

**Figure 3:**
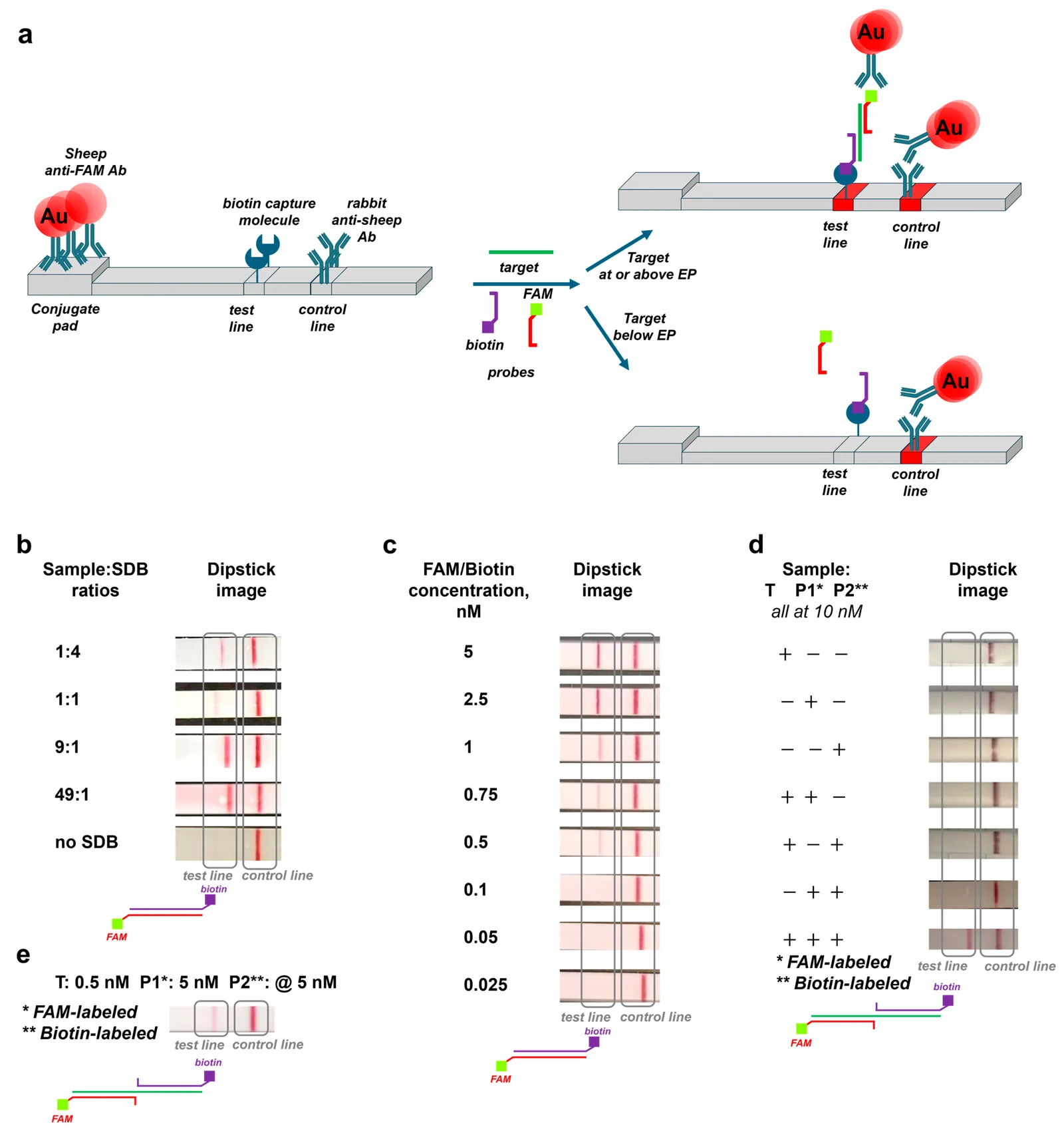
(**a**) Schematics of LFA device. (**b**) Evaluation of sample/sample dilution buffer (SDB) ratios. A FAM-biotin-labeled duplex (20 nM) was diluted with SDB at the indicated ratios and mixed with running buffer. Non-cropped images of dipsticks are included in Figure S2. (**c**) The lowest detectable amount of biotin-/FAM-containing hybrid at approx. 0.5 nM. Solutions of FAM-/biotin-labeled duplex were diluted with hybridization buffer to yield the indicated concentration and then mixed with SDB and running buffer. Non-cropped images of dipsticks are included in Figure S3. (**d**) For specificity evaluation, solutions of the target, FAM-labeled probe, biotin-labeled probe, target–probe duplexes, probe mixture, and mixture containing the target and both probes were evaluated with LFA dipsticks. All strands were prepared in hybridization buffer at 10 nM, diluted with SDB, and mixed with the running buffer. Non-cropped images of dipsticks are included in Figure S4. (**e**) FAM-/biotin-labeled TPP at 0.5 nM level is visible in the LFA. An uncut image of the dipstick is included in Figure S5.

**Figure 4:**
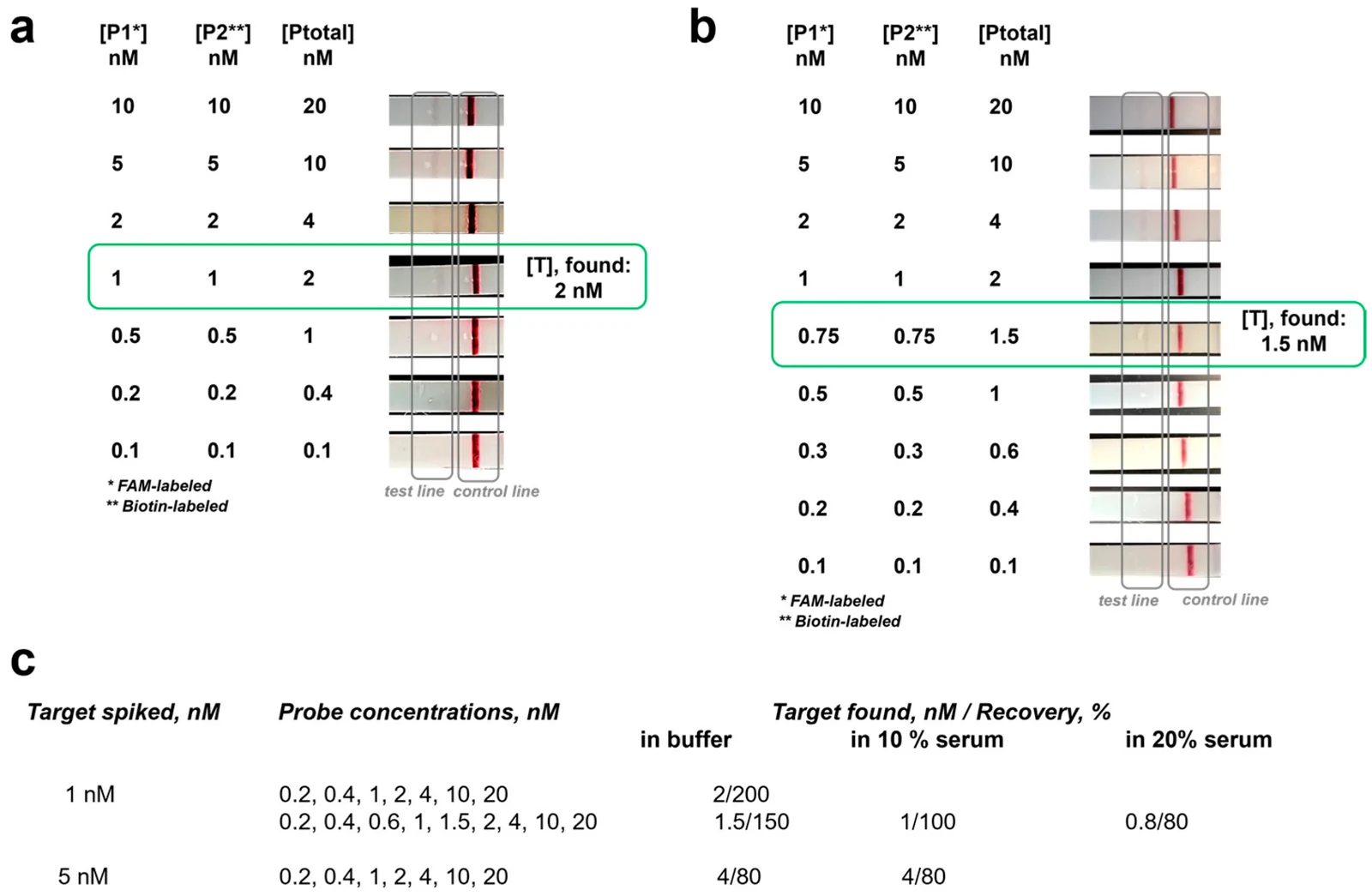
Accuracy of Y/N DNA target analysis on lateral flow strips. (**a**,**b**) Analysis of 1 nM target with series of probes with different steps between concentrations. Additional images are included in Figures S5–S9 and S11–S13. (**c**) Summary of quantitative data. Each experiment was repeated 2–4 times with no variation between replicates (except 20% serum, Figure S13).

Before LFA analysis, hybridized samples were diluted with sample dilution buffer (SDB) and running buffer. Typically, a 45 μL aliquot was diluted with 5 μL of SDB and mixed with 100 μL of running buffer in a 0.6 mL centrifuge tube. LFA dipsticks were dipped into the centrifuge tubes; after 20 min, their images were taken with a cell phone. For Figure 3b–d, images were cropped to focus on the test and control lines. For Figure 3e and Figure 4a,b, images were cropped to focus on the test and control lines, and the brightness/contrast of each strip was uniformly adjusted in PowerPoint. The parameters were adjusted for the whole image; no parts were cut or replaced. All original, unmodified images are included in the Supporting Information (Figures S2–S9).

*Sample dilution buffer/sample dilution ratio*. To establish an optimal SDB/sample ratio (Figure 3b and Figure S2), FAM- and biotin-labeled duplex strands (both at 20 nM) were mixed in hybridization buffer and incubated for 2 h at room temperature; then, samples were diluted with SDB at the ratios indicated in Figure 3b to yield a total volume of 50 μL. The SDB-diluted sample (50 μL) was mixed with 100 μL of running buffer (supplied with the LFA kit) and analyzed via a dipstick.

*Lowest detectable concentration of biotin/FAM hybrid.* To determine the lowest detectable concentration of the FAM/biotin hybrid (Figure 3c and Figure S3), FAM- and biotin-labeled duplex strands (both at 50 nM) were mixed and incubated for 2 h at room temperature. A series of dilutions with hybridization buffer to yield samples at the concentrations indicated in Figure 3c were performed. The diluted samples were mixed with SDB and running buffers and analyzed with LFA dipsticks.

*Selectivity.* The following samples were prepared to evaluate assay selectivity (all at 10 nM): target, biotin-labeled probe 1, FAM-labeled probe 2, target/right arm (FAM-labeled probe 2) duplex, target/left arm (biotin-labeled probe 1) duplex, mixture of FAM- and biotin-labeled probes 2 and 1, sample containing target and both probes (TPP). Each sample was denatured at 95 °C for 5 min, cooled overnight to room temperature, and diluted with SDB and running buffer before analysis with LFA dipsticks (Figure 3d and Figure S4).

*Stoichiometric quantitative analysis with LFA.* To assess the system’s quantitative capability, targets at 0.1 nM, 1 nM, 5 nM, or 10 nM were spiked with probe concentrations ranging from 0.1 to 10 nM (total probe concentration; probes are at equimolar amounts). After 30–120 min of incubation, samples were analyzed using LFAs. The recoveries were calculated as the % ratio of the “spiked” amount to the “found” one (first threshold level with visible test line).

*Serum compatibility experiments.* To evaluate assay performance in biologically relevant environments, the hybridization buffer was supplemented with 10% or 20% serum at the target–probe isothermal mixing step.

## 3. Results

### 3.1. Engineering Negative Cooperativity into Nucleic Acid Hybridization

To engineer negative cooperativity into the stoichiometric analysis of oligonucleotides, we designed two probes, P1 (right arm) and P2 (left arm), that are complementary to adjacent domains on a target T but incorporate “interfering overlaps” (Figure 1c). When either probe binds the target with affinity *K*_1_, duplex TP1 or TP2 is formed (for simplicity, called TP). The overlap region in TP1 or TP2 weakens the affinity towards the second binding event, characterized by constant *K*_2_. Therefore, below the equivalence point, TPP does not appreciably form; however, at and above the equivalence point, TPP forms with an affinity of *K*_2_. By design, *K*_2_ ≪ *K*_1_. Importantly, our data (*vide infra*) indicate that the model is tolerant to some asymmetry in terms of the slightly different affinities of two probes.

In our previous work, we incorporated NC into target–probe binding and assessed the response profiles to find the equivalence point [41,42]; in this work, we establish the overlap parameters that yield a response closest to Y/N. To achieve the binary (Y/N) behavior, we deliberately varied the probe affinities (*K*s) and the extent of negative cooperativity (*K*_1_/*K*_2_). To generate the ranges of *K*_1_, *K*_2_, and *K*_1_/*K*_2_, we manipulated the probes’ lengths and the magnitude of overlap (Figure 2a, Table S1).

To assess the correlation between *K*, *K*_1_/*K*_2_, and the binary response, we monitored TPP in samples below the equivalence point (10 nM target + 4 nM P1 + 4 nM P2, sample 10:4:4) and above the equivalence point (10 nM target + 15 nM P1 + 15 nM P2, sample 10:15:15) via size exclusion chromatography (SEC) (Figure 2b and Figure S1). The assessment criteria were as follows (Figure 2c): no TPP detected below the equivalence point (i.e., sample 10:4:4) and TPP detected above the equivalence point (i.e., sample 10:15:15). The SEC data indicate that only System 1 satisfies the requirements. We hypothesized that the interference was too substantial in System 2 (*K*_1_/*K*_2_ ~ 10^7^–10^9^) to produce an appreciable TPP quantity, while the *K*_2_ values were lower in System 3.

### 3.2. LFA Design and Performance

For the LFA platform, we deliberately selected generic, commercially available dipsticks that were modified with a biotin capture molecule on the test line, anti-sheep IgG on the control line, and anti-FAM antibody-conjugated gold nanoparticles loaded in the conjugate pad (Figure 3a). We acquired our dipsticks from Cytodiagnostics; however, similar formats are available from a range of suppliers around the world [43]. Therefore, they do not require any fabrication/refabrication to adapt to new pathogens.

In our LFA format, a target should have two labels, biotin and FAM, in order to be captured and visualized; therefore, to interface the stoichiometric assay with the LFA dipsticks, we labeled System 1’s probes with FAM (right arm) and biotin (left arm). In this configuration, only TPP has both labels and is therefore captured and visualized in the test line. Assuming that TPP forms at or above an equivalence point (Figure 1b), the detection of the test line indicates that the equivalence point has been reached.

To establish the optimal sample composition, we evaluated different sample/sample dilution buffer (SDB) ratios to find conditions that (i) minimized original sample dilution while (ii) providing adequate signal readability. SDB is a proprietary formulation supplied with the dipsticks. Upon testing the DNA duplex labeled with FAM and biotin (Figure 3b and Figure S2), we observed clear test bands for sample/SDB ratios of 9:1 to 49:1. Interestingly, no test line was visible for the undiluted sample, indicating the need for SDB. Due to the unknown (to users) composition of SDB, we do not have an explanation for this effect and merely speculate that potentially present surfactants and/or the ionic strength improved lateral migration and antibody–antigen interaction. We proceeded with a 9:1 dilution ratio in all further evaluations.

Next, to establish the analytical merits of our quantitative platform, we determined the lowest detectable amount of the FAM/biotin duplex (Figure 3c and Figure S3). The band for 0.5 nM and above is clearly visible. Furthermore, to confirm the ability to detect 0.5 nM TPP, we “saturated” the 0.5 nM target with 5 nM probes; these conditions support TPP formation. As expected, TPP produced a clearly visible band in the test line (Figure 3e and Figure S3). As the specificity studies (vide supra) demonstrate, the probes did not associate to form FAM- and biotin-containing samples.

Furthermore, to establish the approach’s specificity, we analyzed 10 nM concentrations of all individual components (probes, target), 10 nM target/10 nM probe duplexes, and 10 nM/10 nM/10 nM TPP. A clear band in the test line was observed only when all three components were present (Figure 3d and Figure S4).

### 3.3. Y/N Quantitative Analysis with LFA Dipsticks

Next, we evaluated the stoichiometric LFA for 0.1nM, 1 nM, 5 nM, and 10 nM targets. To assess the approach’s accuracy, we exposed the target-spiked sample to a series of probe solutions containing equimolar levels of biotin- and FAM-labeled probes. After isothermal room-temperature equilibration, we submerged the dipsticks and read the test lines. We define the “found” target concentration as the total probe concentration that produces a visible test line. An initial semi-quantitative screening returned 80–200% recoveries (determined as the ratio of “found” to “spiked”, Figure 4 and Figures S6, S7, S10 and S11). As expected from the results of the lowest detectable amount studies (above), no product was detected for the 0.1 nM target (Figure S12).

Repeated experiments (Figure 4 and Figures S6 and S7) demonstrated no differences between replicates (at least three replicates for 1 nM, 5 nM, and 10 nM target levels in buffer). However, at this point, we hesitate to employ the traditional figures of merit (i.e., standard deviation and relative standard deviation) to characterize the threshold-based results.

Importantly, our platform can be adjusted to better resolve the target quantities. Thus, to tune the assay resolution, we modified the probe solution step frequency. As expected, at a higher number of threshold levels, target quantities were established with improved accuracy (150% vs. 200%, Figure 4). We speculate that more frequent probe concentration will further improve the resolution.

Subsequently, to demonstrate the system’s ability to operate in complex biological backgrounds, we analyzed the target in the presence of 10% and 20% serum. The observed recoveries (80–100%) are consistent with those obtained in the buffer experiments conducted at the same probe concentrations and step frequencies (Figures S8, S9 and S13). The result indicates that nucleases intrinsic to the serum do not influence our platform’s performance within the time limits necessary for the analysis.

## 4. Discussion

Calibration-based methods remain the gold standard for the quantitative analysis of biomolecules. Thus, qPCR and ELISA address the need for the accurate and reliable assessment of oligonucleotides and proteins, respectively, in a wide range of circumstances, from cutting-edge research to routine healthcare diagnostics. Calibrations are indispensable but do not provide a clear, binary Y/N readout. Simplicity of interpretation is vital for platforms that aim for diagnostic accessibility.

In this work, we have established a pathway towards the Y/N quantitative analysis of oligonucleotide biomarkers. The threshold-based semi-quantitative platform is based on stoichiometry and operates through a binding mechanism involving negative cooperativity in probe–target interactions. We established binding model characteristics that support the Y/N capability.

Furthermore, we interfaced the stoichiometric platform with a universal lateral flow format, bringing it closer to REASSURED compliance. We demonstrate that the system provides accurate Y/N semi-quantitative results for low-nanomolar (i.e., 1 nM) quantities of oligonucleotide targets without requiring any amplification steps. The sensitivity exceeds [20,22,23,24] or is on par with that of reported quantitative/semi-quantitative equipment-free platforms [21]. However, in contrast to prior reports, our approach does not require any precise strip fabrication and is easily amenable to adjusting the number and range of threshold levels to suit a targeted application. Most up-to-date semi-quantitative threshold-based platforms are usually limited to three threshold points (low, mid-range, high).

Indeed, a key feature of our system is its multi-level tunability. Thus, for tunability with respect to the target, the probes can be re-engineered to target different DNA or RNA sequences without requiring any hardware modifications—a capability that is critical for addressing emerging and evolving pathogens in epidemic or pandemic settings. Beyond target flexibility, the quantitative resolution can be tailored by adjusting the probe concentration range to meet specific application requirements. Thus, our results (Figure 4) emphasize that increasing the probe threshold frequency improves the assay resolution (from 200% to 150% for a 1 nM target). We hypothesize that, if practically meaningful, the resolution may be improved further by narrowing the range and number of threshold levels. However, while improving the resolution is appealing, too many threshold points may make the approach more complex.

As a threshold-based method, our approach is “semi-quantitative”. A biomarker-specific quantitative resolution will define a range and number of thresholds. If a high resolution is required, a realistic protocol for an unknown target may involve two steps: e.g., a wide-range screening to estimate a target quantity followed by a more detailed evaluation if necessary. As an example, a single set of threshold levels was used for the preliminary evaluation of three target levels, 1 nM (Figure 4a), 5 nM (Figure S7), and 10 nM (Figure S11). Then, a more accurate result is demonstrated for a 1 nM target after a more detailed evaluation (Figure 4b). While the envisioned protocol may appear to introduce complexity, the ease of use (equipment-free) and interpretation (Y/N) will not be compromised. We also foresee no significant changes in analysis speed: eventually, multi-lane LFAs or multi-well reagent packs with individual dipsticks can be used in parallel [44,45].

Overall, our approach enables the detection of a stoichiometric equivalence point using simple, generic, commercially available lateral flow dipsticks, which are widely recognized as simple, robust, and highly effective analytical tools. The reported system achieves low-nanomolar quantitation limits comparable to those of previously reported quantitative LFAs [25]; however, our platform enables an easy-to-interpret Y/N readout without any reading device. The majority of reported quantitative/semi-quantitative LFAs either involve the precise deposition of recognition elements at the fabrication stage [20,21,22,23,24] and/or (ii) signal readout device or software [19,21,24,25,26,27,28,29,30,31]. Reliance on generic FAM-/biotin-based strips further streamlines assay development for other pathogens, as only the reagent set must be adjusted for different targets.

For the platform’s future proliferation, some essential areas will need to be addressed. For example, our sub-nanomolar lowest detectable amounts exceed the expected levels of most nucleic acid biomarkers. To address this deficiency, we are currently exploring the integration of stoichiometric activation with isothermal amplification. With upfront amplification, we expect to reduce the lowest detectable concentrations to practically meaningful levels. Furthermore, a viable platform must bind targets that are likely to be folded into secondary structures. To enable proper binding, unfolding functionalities might be incorporated [46]. Moreover, in the current format, our platform does not support the analysis of dsDNA targets; some redevelopments will be necessary to address the quantitative analysis of these.

## Data Availability

The original contributions presented in this study are included in the article/Supplementary Materials. Further inquiries can be directed to the corresponding author.

## Supplementary Materials

The following supporting information can be downloaded at https://www.mdpi.com/article/10.3390/bios16090465/s1. Table S1: Oligonucleotide sequences. Figure S1: SEC chromatograms. Figure S2: Evaluation of Sample:SDB dilution ratios. Figure S3: Evaluation of the Limit of Detection. Figure S4: Evaluation of specificity towards system components. Figure S5: Detection of TPP at LOD level. Figure S6: Target quantitation with LFA: 1 nM, buffer. Figure S7: Target quantitation with LFA: 5 nM, buffer. Figure S8: Target quantitation with LFA: 5 nM, 10 % serum. Figure S9: Target quantitation with LFA: 1 nM, 10 % serum. Figure S10: PAGE separation of System 1 components. Figure S11: Target quantitation with LFA: 10 nM, buffer. Figure S12: Target quantitation with LFA: 0.1 nM, buffer. Figure S13: Target quantitation with LFA: 1 nM, 20 % serum.

## Abbreviations

The following abbreviations are used in this manuscript:

FAM	Carboxyfluorescein, fluorescein amididate
SEC	Size exclusion chromatography
NC	Negative cooperativity
SDB	Sample dilution buffer
Y/N	Yes/no
REASSURED	Real-time, ease of specimen collection, affordable, sensitive, specific, user-friendly, robust and rapid, equipment-free, deliverable
DNA	Deoxyribonucleic acid
RNA	Ribonucleic acid
LFA	Lateral flow assay
ssDNA	Single-stranded DNA
dsDNA	Double-stranded DNA
