# Supplementary material for: Yes/No Quantitative Analysis of Single-Stranded Oligonucleotides with Lateral Flow Assays
Source: Biosensors (Basel). 2026 Aug 26;16(9):465. doi: 10.3390/bios16090465 (PMC13604401; doi:10.3390/bios16090465)
Supplement: Supplementary file 1 [file biosensors-16-00465-s001.zip › biosensors-4478768-supplementary.pdf]

Yes/No Quantitative Analysis of Single-Stranded Oligonucleotides with Lateral Flow Assays

*Niusha Hassandoost, Leslie Munoz, Kerrigan Kotecki, and Irina V. Nesterova**

Department of Chemistry and Biochemistry, Northern Illinois University, DeKalb, IL 60115, USA

ELECTRONIC SUPPORTING INFORMATION

TABLE OF CONTENTS:

Table S1	Oligonucleotide sequences	p. ESI-2
Figure S1	SEC chromatograms	p. ESI-3
Figure S2	Evaluation of Sample:SDB dilution ratios	p. ESI-4
Figure S3	Evaluation of the Limit of Detection	p. ESI-4
Figure S4	Evaluation of specificity towards system components	p. ESI-5
Figure S5	Detection of TPP at LOD level	p. ESI-5
Figure S6	Target quantitation with LFA: 1 nM, buffer	p. ESI-6
Figure S7	Target quantitation with LFA: 5 nM, buffer	p. ESI-7
Figure S8	Target quantitation with LFA: 5 nM, 10 % serum	p. ESI-8
Figure S9	Target quantitation with LFA: 1 nM, 10 % serum	p. ESI-9
Figure S10	PAGE separation of System 1 components	p. ESI-10
Figure S11	Target quantitation with LFA: 10 nM, buffer	p. ESI-10
Figure S12	Target quantitation with LFA: 0.1 nM, buffer	pESI-11
Figure S13	Target quantitation with LFA: 1 nM, 20 % serum	pESI-12

Table S1. Oligonucleotide sequences

**Sequence name and pictogram**	**Sequence, 5′ → 3′**	$\Delta G, kcal/mol$ K_1*	$\Delta G, kcal/mol$ K_2**	K_1/K_2***
Target	TAT TTC TTA TTC ATA TTC AGG CCG ACA TCG AGA CCA GGG CAC GC			
**System 1**				
Probe 1 (left arm) or (biotin-modified)†	GAT GTC GGC CTG AAT ATG AAT AAG AAA TA	-51.14 3.1×10^{37}	-46.14 6.6×10^{33}	5×10^{3}
Probe 2 (right arm) or (FAM-modified)‡	GCG TGC CCT GGT CTC GAT GTC	-43.57 8.7×10^{31}	-38.7 2.3×10^{28}	4×10^{3}
**System 2**				
Probe 1 (left arm)	TC GAT GTC GGC CTG AAT ATG AAT AAG AAA TA	-56.33 2.0×10^{41}	-46.14 6.6×10^{33}	3×10^{7}
Probe 2 (right arm)	GCG TGC CCT GGT CTC GAT GTC GG	-50.25 6.8×10^{36}	-38.7 2.3×10^{28}	3×10^{8}
**System 3**				
Probe 1 (left arm)	GAT GTC GGC CTG AAT AT	-31.79 2.0×10^{23}	ΔG = -26.79 4.3×10^{-19}	5×10^{3}
Probe 2 (right arm)	TGC CCT GGT CTC GAT GTC	-35.48 1.0×10^{26}	-30.60 2.7×10^{22}	4×10^{3}
**Duplex**				
5′ Biotin-labeled duplex strand*	TATTTCTTATTCATATTCAGGAAGACAT			
5′ FAM-labeled duplex strand‡	ATGTCTTCCTGAATATGAATAAGAAATA			

* calculated as $K = e^{\frac{-\Delta G}{RT}}$ using ΔG values for probe/target complement obtained by OligoAnalyzer tool

** calculated as $K = e^{\frac{-\Delta G}{RT}}$ using ΔG values for probe/target complement with ½ overlap region excluded, obtained by OligoAnalyzer tool

† modified with biotin at 3′ end (IDT"s code /3Bio/)

‡ modified with FAM at 5′ end (IDT's code /56-FAM/)

* modified with biotin at 5′ end (IDT's code /5Biosg/)

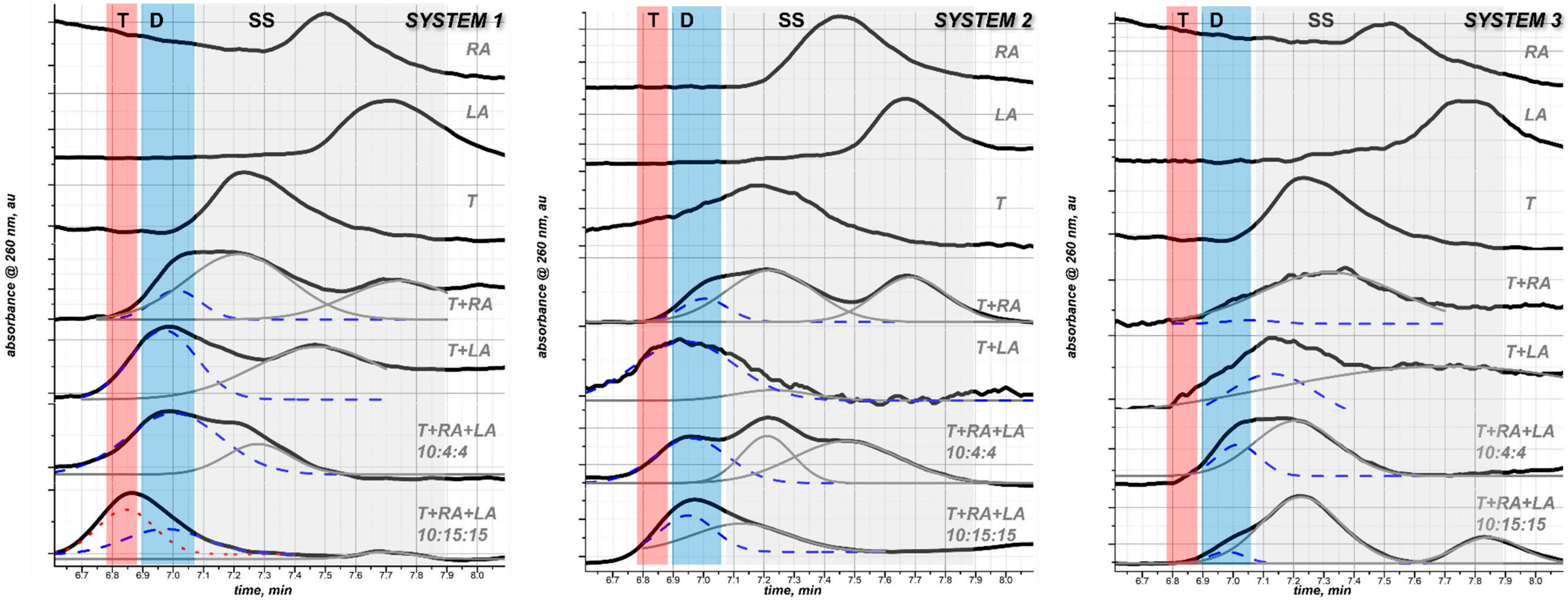

Figure S1. SEC chromatograms of single strands (SS: target at 10 nM (T), right arm probe at 10 nM (RA), left arm probe at 10 nM (LA)), duplexes (T+RA, T+RA, all strands at 10 nM), and samples containing 10 nM target with 4 nM of each probe (10:4:4) and with 15 nM of each probe (10:15:15).

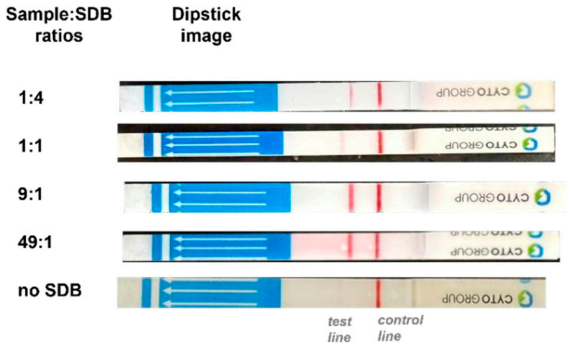

Figure S2. Dipstick images for evaluation of Sample: SDB dilution ratios. FAM/Biotin-modified duplex strands at 10 nM each were allowed to hybridize at room temperature. The hybridized samples were diluted with SDB at the indicated ratios and mixed with 2 volumes of running buffer.

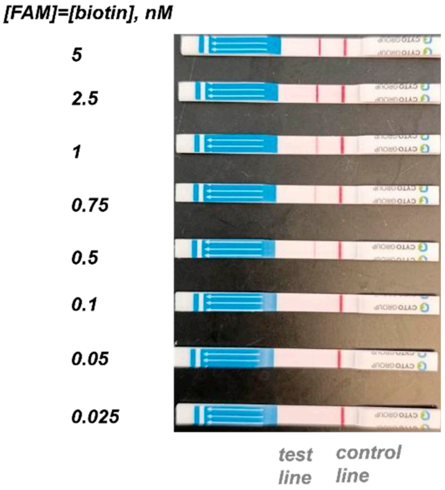

Figure S3 Dipstick images for evaluation of the lowest detectable concentration of FAM/Biotin hybrid. FAM/Biotin-modified duplex strands at 10 nM each were allowed to hybridize at room temperature. The hybridized samples were diluted with SDB at 9:1 ratio and mixed with 2 volumes of running buffer.

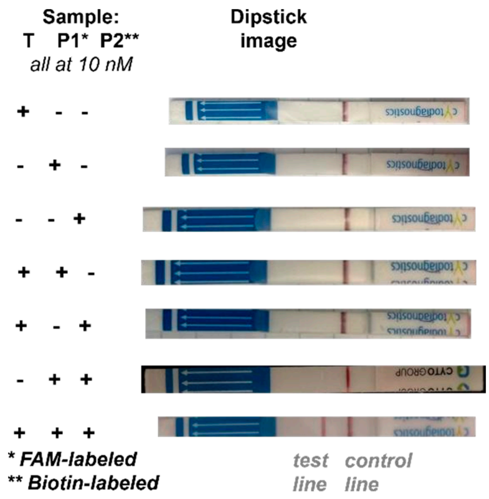

Figure S4. All System 1's components at 10 nM were allowed to hybridize at room temperature, diluted with SDB at 9:1 ratio, and mixed with 2 volumes of running buffer. Probes modified with biotin and FAM were used in the experiments.

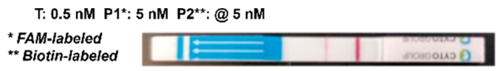

Figure S5. Detection of TPP at lowest detectable concentration of FAM/Biotin hybrid. Target and two System 1's modified probes were allowed to hybridize at room temperature, diluted with SDB at 9:1 ratio, and mixed with 2 volumes of running buffer.

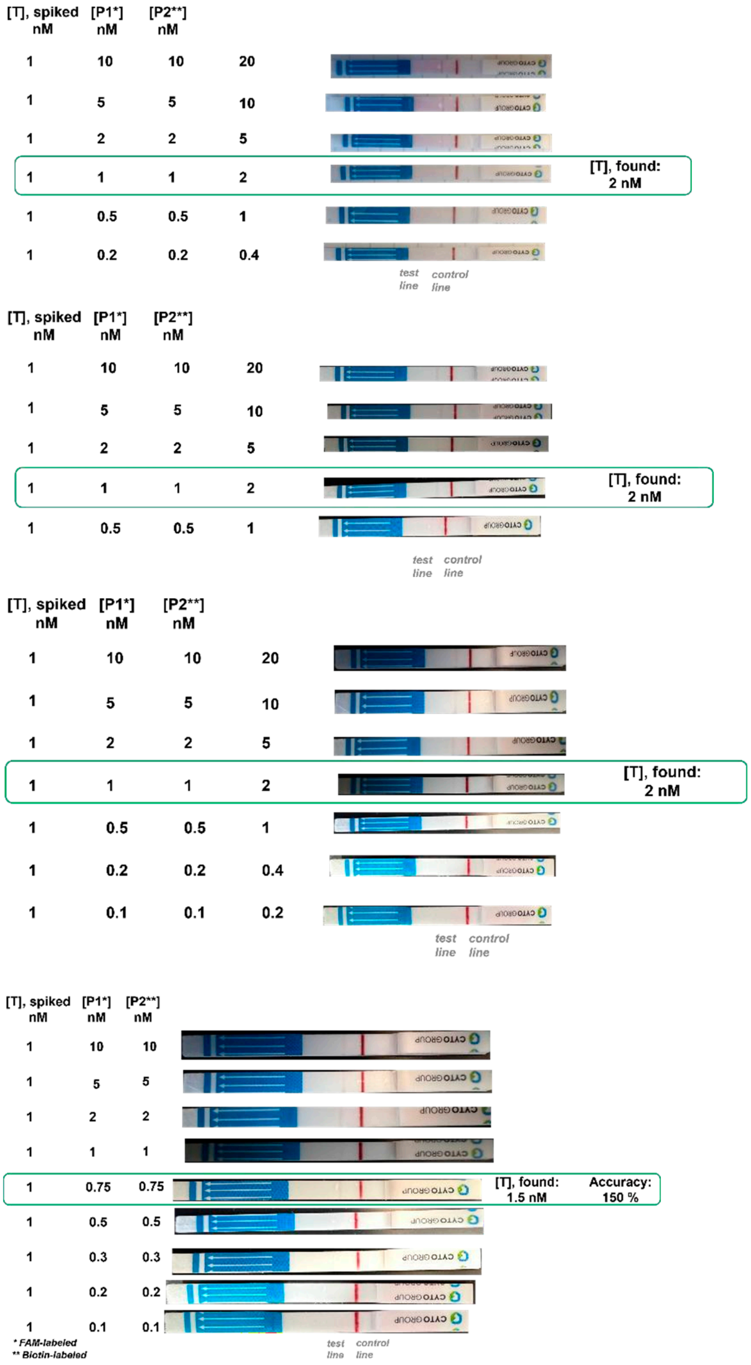

Figure S6. Analysis of 1 nM samples in hybridization buffer. Target and two System 1's modified probes were allowed to hybridize at room temperature, diluted with SDB at 9:1 ratio, and mixed with 2 volumes of running buffer.

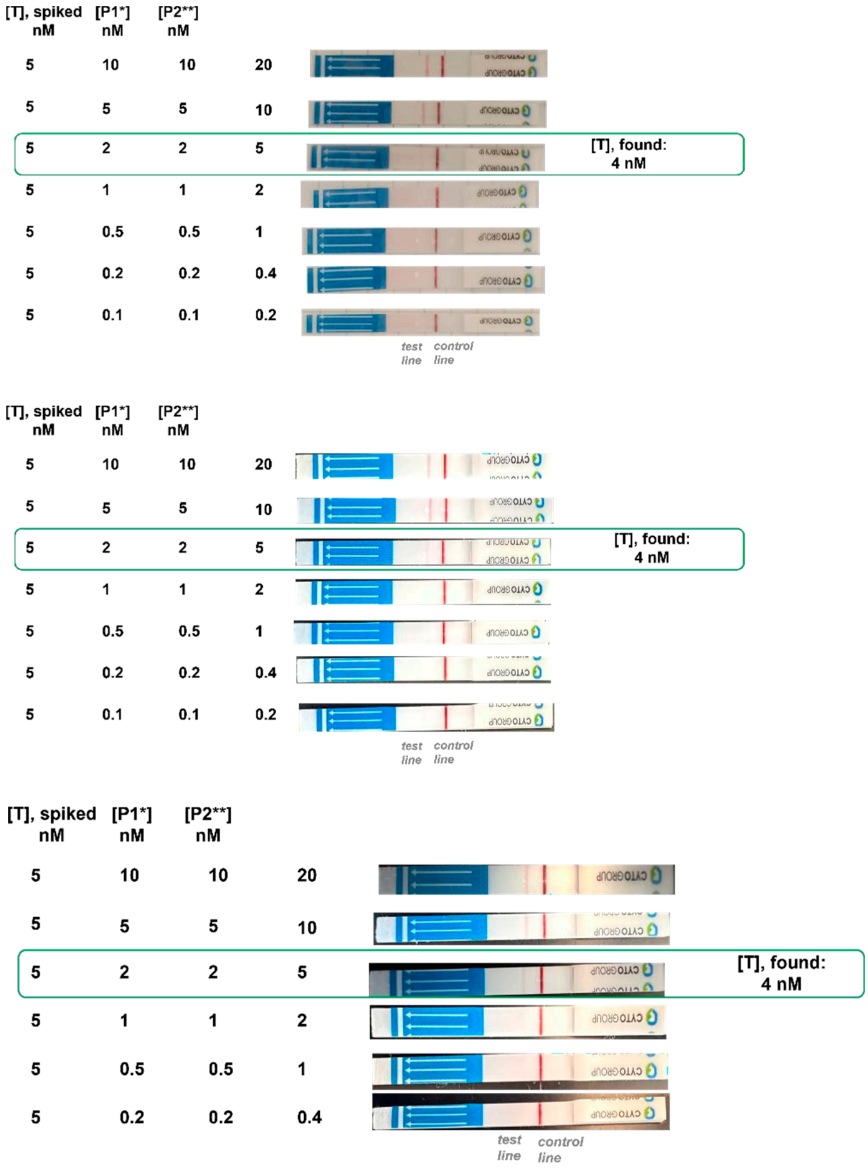

Figure S7. Analysis of 5 nM samples in buffer. Target and two System 1's modified probes were allowed to hybridize at room temperature, diluted with SDB at 9:1 ratio, and mixed with 2 volumes of running buffer.

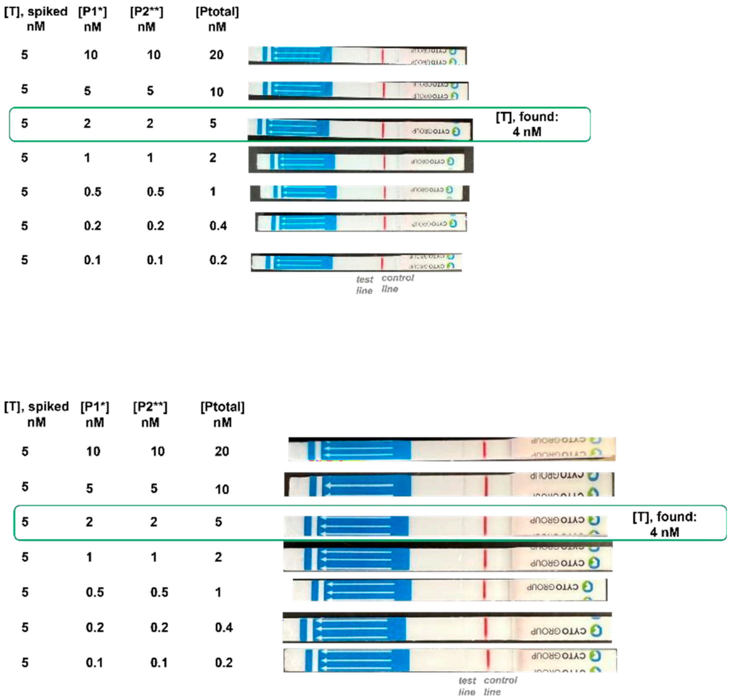

Figure S8. Analysis of 5 nM samples in the presence of 10 % serum. Target and two System 1's modified probes were allowed to hybridize in hybridization buffer supplemented with 10 % serum at room temperature, diluted with SDB at 9:1 ratio, and mixed with 2 volumes of running buffer.

[T], spiked nM	[P1*] nM	[P2**] nM	[Ptotal] nM
1	10	10	20
1	5	5	10
1	2	2	5
1	1	1	2
1	0.75	0.75	1.5
1	0.5	0.5	1
1	0.3	0.3	0.6
1	0.2	0.2	0.4
1	0.1	0.1	0.2

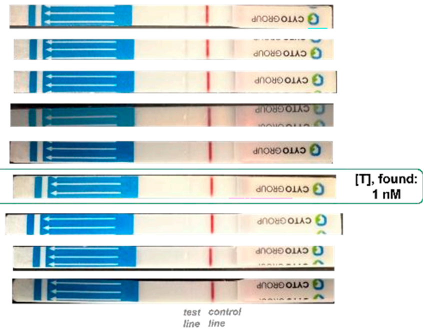

[T], spiked nM	[P1*] nM	[P2**] nM	[Ptotal] nM
1	10	10	20
1	5	5	10
1	2	2	5
1	1	1	2
1	0.75	0.75	1.5
1	0.5	0.5	1
1	0.3	0.3	0.6
1	0.2	0.2	0.4
1	0.1	0.1	0.2

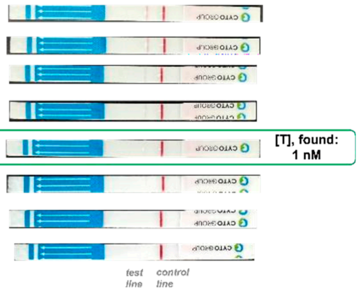

Figure S9. Analysis of 1 nM samples in the presence of 10 % serum. Target and two System 1's modified probes were allowed to hybridize in hybridization buffer supplemented with 10 % serum at room temperature, diluted with SDB at 9:1 ratio, and mixed with 2 volumes of running buffer.

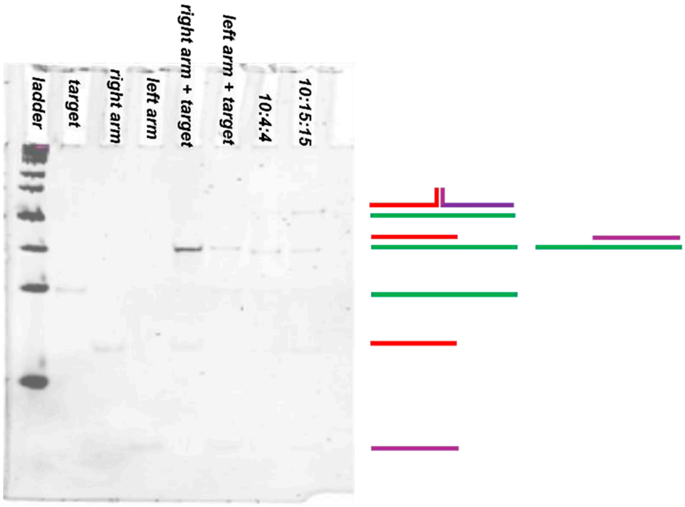

Figure S10. Separation of System 1 components at 20 % native polyacrylamide gel electrophoresis. Separation conditions: 0.5 × TBE, 150 V, 3 h, 4 ºC. All components are at 10 nM except samples 10:4:4 (10 nM target, 4 nM right arm, 4 nM left arm) and 10:15:15 (10 nM target, 15 nM right arm, 15 nM left arm).

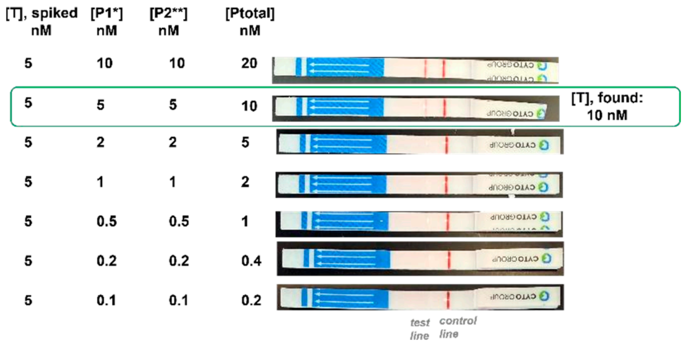

Figure S11. Analysis of 10 nM sample in buffer. Target and two System 1's modified probes were allowed to hybridize at room temperature, diluted with SDB at 9:1 ratio, and mixed with 2 volumes of running buffer.

[T], spiked nM	[P1*] nM	[P2**] nM	[Ptotal] nM
0.1	2	2	4
0.1	1	1	2
0.1	0.5	0.5	1
0.1	0.2	0.2	0.4
0.1	0.1	0.1	0.2
0.1	0.05	0.05	0.1
0.1	0.02	0.02	0.04

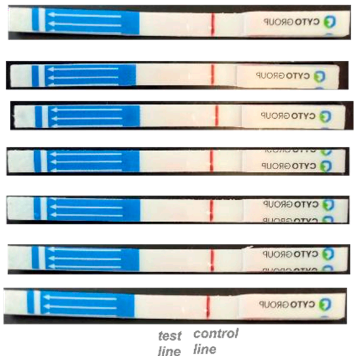

Figure S12. Analysis of 0.1 nM sample in buffer. Target and two System 1's modified probes were allowed to hybridize at room temperature, diluted with SDB at 9:1 ratio, and mixed with 2 volumes of running buffer.

[T], spiked nM	[P1*] nM	[P2**] nM	[Ptotal] nM
1	10	10	20
1	5	5	10
1	2	2	4
1	1	1	2
1	0.75	0.75	1.5
1	0.5	0.5	1
1	0.3	0.3	0.6
1	0.2	0.2	0.4
1	0.1	0.1	0.2

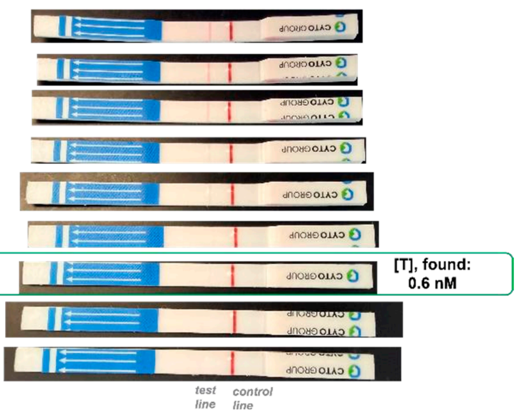

[T], spiked nM	[P1*] nM	[P2**] nM	[Ptotal] nM
1	10	10	20
1	5	5	10
1	2	2	4
1	1	1	2
1	0.75	0.75	1.5
1	0.5	0.5	1
1	0.3	0.3	0.6
1	0.2	0.2	0.4
1	0.1	0.1	0.2

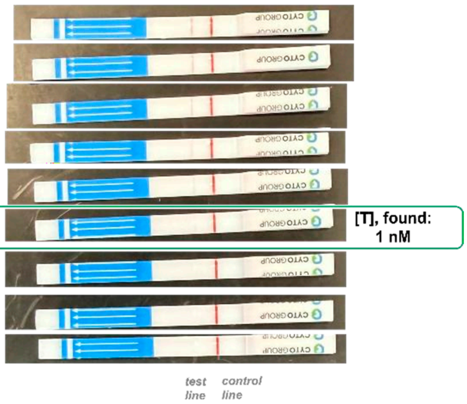

Figure S13. Analysis of 1 nM sample in 20% serum/80 % buffer. Target and two System 1's modified probes were allowed to hybridize at room temperature, diluted with SDB at 9:1 ratio, and mixed with 2 volumes of running buffer. The reported recovery of 80 % (Figure 4c) is calculated as an average of two replicates (0.6 nM and 1 nM).
